# The Degradation Interface of Magnesium Based Alloys in Direct Contact with Human Primary Osteoblast Cells

**DOI:** 10.1371/journal.pone.0157874

**Published:** 2016-06-21

**Authors:** Nezha Ahmad Agha, Regine Willumeit-Römer, Daniel Laipple, Bérengère Luthringer, Frank Feyerabend

**Affiliations:** 1 Division of Metallic Biomaterials, Institute of Material Research, Helmholtz-Zentrum Geesthacht, Geesthacht, Germany; 2 Division of Materials Physics, Institute of Material Research, Helmholtz-Zentrum Geesthacht, Geesthacht, Germany; North Carolina A&T State University, UNITED STATES

## Abstract

Magnesium alloys have been identified as a new generation material of orthopaedic implants. *In vitro* setups mimicking physiological conditions are promising for material / degradation analysis prior to *in vivo* studies however the direct influence of cell on the degradation mechanism has never been investigated. For the first time, the direct, active, influence of human primary osteoblasts on magnesium-based materials (pure magnesium, Mg-2Ag and Mg-10Gd alloys) is studied for up to 14 days. Several parameters such as composition of the degradation interface (directly beneath the cells) are analysed with a scanning electron microscope equipped with energy dispersive X-ray and focused ion beam. Furthermore, influence of the materials on cell metabolism is examined via different parameters like active mineralisation process. The results are highlighting the influences of the selected alloying element on the initial cells metabolic activity.

## Introduction

Due to its inherent degradation property, magnesium (Mg) based alloys are a new generation material of orthopaedic implants eliminating further complications that may accompany retrieval surgery and can be especially helpful in bone fixation for children. In addition to their degradability, some magnesium alloys have been proven to exhibit good bioactivity as well as physical and mechanical properties comparable to those of bone [1–3]. Selecting smart alloying elements which can deliver multi functionality of the designed magnesium alloy inspired the motivation of many studies. For instance, silver (Ag) is known for its antibacterial effects, and its implementation in different biomedical applications has been widely investigated [4–6]. In a binary alloying system, adding 2 wt. % silver to a magnesium cast followed by solution (T4) heat treatment led to acceptable level of corrosion resistance and improved the mechanical properties of the cast [7].

Alloying magnesium with rare earth elements (REEs) to improve its properties is also being investigated. For example, one systematic study examined the addition of gadolinium (Gd), a rare earth element, to magnesium to generate Mg-XGd binary systems, where X was 2, 5, 10, and 15 wt. % [8]. This study showed that increasing the Gd content improved the mechanical properties, but the addition of this alloying element should coincide with the corrosion behaviour because the improved corrosion resistance was obtained in the range of 5–10 wt. % of gadolinium addition. Furthermore, many studies reported an anticarcinogenic effect of Gd [9–12].

The use of magnesium as an orthopaedic implant is under investigation worldwide. *In vitro* studies show that many parameters and factors can influence magnesium implant degradation, such as the inorganic salts that contribute to the physiological solutions [13–16], proteins [17, 18], pH, and other parameters concerning the buffering effect, such as CO_2_ and bicarbonate salts [19]. Therefore, simulating a real physiological environment is not an easy approach, and including the cell interaction in the studied system introduces further complexity. Many *in vitro* studies have focused on studying the influence of magnesium degradation on the cells using either extract or a highly dissolved salt of Mg [20–25]. Other studies have focused on the influence of the new alloying system or surface coating and treatment on cell adherence, activity, and cytotoxicity [26–28]. However, fewer studies analysed the interface between the cells and the degraded material and the consequent influence of the cells on the interface composition. In this study, we thoroughly investigated the degradation interface of pure Mg (used as a control) and two alloys: Mg-2Ag and Mg-10Gd.

Primary human osteoblasts were selected for this study because (I) they play an essential role in bone formation by inducing and regulating the mineralization of the extracellular matrix (i.e., hydroxyapatite (HA) crystal formation) [29, 30] and (II) they are a better *in vitro* model to mimic *in vivo* than the generally used osteoblast cell lines [31].

## Materials and Methods

### Material Production

The two magnesium alloys (Mg-2Ag, Mg-10Gd) were produced by permanent mould gravity casting. After melting the pure Mg, the melt was held at 720°C, and the preheated alloying elements were added with continuous stirring for 15 minutes. The melt was poured into a preheated (550°C) permanent steel mould treated with Boron Nitride. During the casting process, a cover gas was used (SF_6_ and Ar mixture). The alloys were homogenized with a T4 heat treatment prior to extrusion in an Ar atmosphere at 550°C (Mg-10Gd) and at 420°C (Mg-2Ag) for 6 h. Subsequently, the alloys were indirect extruded at an extrusion ratio of 4/25 (Strangpreßzentrum Berlin, Berlin, Germany). The chamber of the extrusion machine was set to 370°C, and the billets (d = 30 mm) were preheated for one hour at 370°C (Mg-2Ag) and 430°C (Mg-10Gd). The extrusion speed ranged from 3 to 4.5 mm/sec. Pure Mg was cast using a permanent mould direct chill cast. The cast billet (d = 110 mm) was indirect extruded at an extrusion ratio 1/84. The temperature of the billet was 340°C, and the speed of the extrusion was 0.7 mm/sec. The chemical composition was determined by spark emission spectrometry for Mg, iron (Fe), copper (Cu), and nickel (Ni) (Spectrolab M, Spektro, Germany), and the concentrations of Ag and Gd were determined by X-ray fluorescence spectrometry (Bruker AXS S4 Explorer, Bruker AXS GmbH, Germany). Discs (diameter of 10 mm and 1.5 mm thickness) were machined from the extruded bars (Henschel KG, Munich, Germany), and the samples were sterilized using gamma radiation at a dose of 29.2 KGy (BBF GmbH, Stuttgart, Germany).

### Immersion Procedure

Dulbecco’s Modified Eagle’s Medium (DMEM; Life technologies, Darmstadt, Germany) containing 10% Fetal Bovine Serum (FBS; PAA laboratories, Linz, Austria) and 1% Penicillin/Streptomycin (P/S; Invitrogen, Darmstadt, Germany) was used as the cell culture and immersion medium. The samples were pre-incubated for 24 h under cell culture conditions (37°C, 20% O_2_, 5% CO_2_, and 95% relative humidity). The medium was then changed, and the cells were cultured directly on the upper surface of the discs. This protocol has been reported to improve cell adhesion and avoid the strong initial reaction between the material and medium, which reflects in material dissolution, hydrogen evolution and alkalization effect on the surrounding environment. As a consequence of using this protocol, cell stress and death would be avoided [32]. This feature is important in our study, which examined the direct influence of cells on the degradation. Systematically, samples without cells (or ‘‘samples no cells” SNC) were treated in the same manner as samples with cells (SC) by following the same pre-incubation procedure. The SC were pre-incubated and 100 000 human primary osteoblasts were then applied to the top surface and allowed to adhere for 30 min in the incubator (Heraeus, Langenselbold, Germany). Then, medium was added to continue the immersion. In addition, two controls, medium control (MC) and cell control (CC), were added to the experimental layout. Human hip joints were obtained from patients undergoing hip arthroplasty (Hospital Eilbek, Hamburg, Germany; written consent documented) with the approval of the local ethics committee, but without passing patient data (Ethikkommission der Ärztekammer Hamburg, Germany). Cells of at least 5 donors were used. Primary human osteoblasts were isolated according to the protocol of Gallagher [33]. Briefly, cancellous bone was removed from the hip joint bone and transferred to a cell culture flask. Then, the cancellous particles were covered with cell culture medium, incubated until reaching approximately 90% confluence, and divided. For this study, osteoblasts were used up to the third passage.

To clarify the role of osteoblasts in mediating mineralization, L929 mouse fibroblasts cell line (Sigma-Aldrich, Taufkirchen, Germany) was used as a cell control using the same procedure which described above.

### Degradation Rate by Weight Loss Method

Before sample sterilization, the initial weight of the samples as well as the initial pH (SENTRON ARGUS X pH-meter, Fisher Scientific GMBH, Schwerte, Germany) and osmolality (Osmomat 030, Gonotec, Berlin, Germany) of the immersion medium were recorded. Six samples were immersed in 2 mL of immersion medium for total immersion times (with pre-incubation) of 4 and 14 days under cell culture conditions. The immersion medium was changed every 3–4 days to facilitate a semi-static immersion test and prevent saturation effects. The osmolality of the supernatant was measured after each change of the medium. After immersion, the subsequently formed products were removed by treating the corroded disk with chromic acid (180 g/L in distilled water, VWR international, Darmstadt, Germany) for 20 min at room temperature. Images of the samples before and after chromic acid treatment were taken with a Moticam10 instrument (Motic Deutschland GmbH, Wetzlar, Germany). The degradation rate (DR) was calculated in mm/year using the following equation:

$$DR=\frac{8.76\;\times \;10^{4}\;\times \;\Delta g}{A\;\times \;t\;\times \;\rho }$$ (1)

where Δg is the weight change in grams, A is the surface area of the sample in cm^2^, t is the immersion time in hours, and ρ is the density in g/cm^3^.

### Online pH Measurement

The pH was measured continuously throughout the immersion using the SDR SensorDish reader system (PreSens GmbH, Regensburg, Germany). Special 24-well plates were used with an integrated pH sensor (HydroDish, PreSens GmbH, Regensburg, Germany). The measurement method is based on non-invasive luminescence detection. The detectable pH values in this system ranged from five to nine. The experiments were performed by incubating 6 samples of each group (each alloys, with and without cells) in 2 mL of the immersion medium for 14 days. During the immersion and prior to the medium exchanges, the pH values of the supernatants were measured after shaking with (pH-meter Titan X, Fisher Scientific GmbH, Schwerte, Germany) to compare values and ensure the validity of the online pH measurement.

### In Vitro Viability by LIVE/DEAD (Viability/Cytotoxicity) Staining Assay

The cell coverage and viability of osteoblasts were assessed using a LIVE/DEAD (Life technologies, Darmstadt, Germany) assay. To this end, three samples were used from each material that had been immersed for 4 or 14 days. The staining solution was prepared by adding 4 μL Calcein AM (LIVE) and 10 μL Ethidium homodimer-1 (DEAD) to 10 mL of PBS. The samples were first washed with Phosphate Buffered Saline (PBS) solution to eliminate the non-adherent cells, and then immersed in (1.5 mL / sample) of staining solution under cell culture conditions for 20 minutes. The staining solution was then replaced with DMEM, and the samples were visualized by fluorescent microscopy (Nikon, eclipse Ti, Düsseldorf, Germany). The following filters were used: Fluorescein Isothiocyanate; FITC (Ex: 465–495 nm; Em: 515–555 nm; Mirror at 505 nm), and Texas red (Ex: 540–580 nm; Em: 600–660; Mirror at 595 nm).

The cell viability was calculated as follows:

$$\text{Viability }\left(\text{\%}\right)=\frac{{\left(\frac{\text{live cells}}{\text{live cells }+\text{ dead cells }}\right)}_{\text{sample}}}{{\left(\frac{\text{live cells}}{\text{live cells }+\text{ dead cells }}\right)}_{\text{control}}}\;\times 100$$ (2)

### Cell Adhesion and Morphology Determined by Scanning Electron Microscope (SEM)

The SEM samples were fixed overnight in 2.5% glutaraldehyde (Sigma-Aldrich, Steinheim, Germany), and the cells were then incubated with 1% osmium tetroxide (Sigma-Aldrich, Taufkirchen, Germany) for 0.5 h. The samples were then successively dehydrated in 35%, 60%, 80%, and 100% isopropanol, followed by critical point drying (Leica EM CPD030, Wetzlar, Germany) to reach a water-free state. The SEM images were acquired with a Zeiss Ultra 55 (Carl Zeiss GmbH, Oberkochen, Germany) scanning electron microscope at 2 kV using an SE2 –detector.

### Cytotoxicity Assessment by Measuring Lactate Dehydrogenase (LDH)

LDH cytotoxicity detection kit (Roche Diagnostic GmbH, Mannheim, Germany) was used to measure the active LDH released from the damaged cells into the medium based on a colorimetric method. This assay indicates the damage to the plasma membrane of cells induced by the degradation of the studied materials. The measurement was performed after 1, 2, 3, 7, and 14 days of incubation. A standard curve was prepared for each measured 96-well plate to quantify the LDH in the extract. The standard curve was generated by adding L-lactate dehydrogenase (L-LDH) from bovine heart (Sigma-Aldrich, Steinheim, Germany) to the used immersion medium, and this solution was then serially diluted to obtain a gradual series of concentrations. The controls consisted of medium alone and samples without cells. Furthermore, cells alone were used as a “low control” (i.e., low or normal LDH release), and cells with Triton X-100 (Sigma-Aldrich, Steinheim, Germany) were used as a “high control” (i.e., high LDH release) at each measured time point. The assay consisted of adding 100 μL of these samples, standards, and controls to 100 μL of the substrate buffer. The samples were then incubated in the dark at room temperature for 20 minutes, and the absorbance was measured at 490 nm with a reference at 620 nm using a SUNRISE absorbance reader (Tecan GmbH, Grailsheim, Germany).

The cytotoxicity was calculated according to the following formula:

$$Cytotoxicity\;\left(\;\%\right)\;=\frac{experiment\;value\;-\;low\;control}{high\;control-low\;control}\;\times 100$$ (3)

### Double Staining for Bone Mineralization (Osteo-Image) and Cell’s DNA (DAPI)

Osteo-Image (Lonza, Walkersville, USA) was used as a specific stain for hydroxyapatite (HA). Three samples per condition, SC, SNC, CC, and MC, were used to identify the possibility of HA precipitation under CCC and then evaluate the mineralization matrix deposition based on the differentiated osteoblasts on each alloy. The L929 mouse fibroblast was used as a cell control. The staining protocol started with removing the plate from the incubator and allowing it to reach room temperature (RT). The medium was then removed, and the discs were washed once with PBS. The samples were subsequently fixed with 3.7% formaldehyde (Sigma-Aldrich, Taufkirchen, Germany) at RT for 20 minutes and then washed twice with Osteo-Image wash buffer (diluted 1:10 in deionized water). One millilitre of the staining reagent (diluted 1:100 in staining reagent dilution buffer) was added to each sample, and the mixture was incubated at RT for 30 minutes protected from light. Then, the staining reagent was removed, and the samples were washed three times with the wash buffer. The samples were analysed under a fluorescence microscope with a FITC filter. To obtain an image of the entire discs large images (6 x 6) with 4x magnification and 5% overlapping between the acquired images were collected. To visualize the cell-HA distribution, samples with cells and cell control were also stained with DAPI (4´,6-Diamidino-2-Phenylindole Dihydrochloride) (Sigma-Aldrich, Taufkirchen, Germany). Following the protocol, 5 mg of the reagent was first dissolved in 10 mL of doubled distilled H_2_O. Subsequently, 100 μL of this solution was added to 9.9 mL of methanol (Promochem, LGC standards GmbH, Wesel, Germany) to obtain the working solution. Each sample was immersed in 1 mL of the working solution, and incubated for 15 min at 37°C in the dark. The samples were analysed by fluorescence microscopy with a DAPI filter (Ex: 340–380 nm; Em: 435–485 nm; Mirror at 400 nm).

The Osteo-Image staining was evaluated by quantifying the mineralization matrix shown in the large images in monochromatic mode (FITC) using the ImageJ software [34]. First, the images were converted to binary images and then segmented by histogram thresholding to isolate the stained areas. The amount of staining in the image was measured in area %.

### Analysis of the Degradation Interface Using a Scanning Electron Microscope Equipped with Energy Dispersive X-Ray Spectroscopy and a Focused Ion Beam (SEM/EDX/FIB)

Scanning Electron Microscopy (SEM) measurements were conducted using an Auriga microscope (Zeiss, Oberkochen, Germany) equipped with an Energy Dispersive X-ray Spectroscopy (EDX) device (Apollo XP from EDAX, Ametek GmbH, Wiesbaden, Germany). The SEM images were obtained at an accelerating voltage of 2 kV with the SE2—detector. Cross sections of the SC and negative control samples (SNC) were prepared by ion beam milling at 30 keV using a gallium focused ion beam (FIB) attached to the Auriga. To prevent damage to the cells and the corrosion layer as well as to obtain a precise cut along the degradation layer, a layer of platinum was deposited on the surface by the Gas-Injection-System (GIS). Five EDX—line scans were performed directly after FIB milling at 15 keV to define the vertical element distribution of the degradation layer on the cross-sections. The EDX scans were conducted in 8 regions of interest in the respective EDX spectrum, i.e., carbon (C), nitrogen (N), oxygen (O), sodium (Na), Mg, phosphorus (P), sulphur (S) and calcium (Ca), complemented by the alloying elements of the respective sample, i.e., Ag and Gd. Each degradation layer cross section was analysed using this method. To obtain a high counting rate for the EDX analysis, the SEM aperture (120 μm diameter) was used in high-current mode.

### Statistics

The SigmaStat package (Systat software GMBH, Erkrath, Germany; version 11.0) was used for statistical analyses. A standard analysis comparing the conditions for Mg-10Gd and pure Mg was conducted using a one way analysis of variance (ANOVA) on ranks with Dunn’s multiple comparison post hoc test. The significance level was 0.05. Differences between the Mg-2Ag conditions were detected using the Holm-Sidak method with a significance level of 0.001.

### Solubility Calculations

Following simple solubility concepts, the possibility of precipitation was calculated for four different Ca-PO_4_ phases in the immersion medium at a pH of 8.3 (a random specified value) and pCO_2_ = 0.05 atm. The utilized concepts are explained in detail in the references [35–37]. Briefly, the ionic strength of the immersion medium was calculated and used to estimate the activity of the specified anions and cations according to the extended Debye-Hückel formula for ionic activity, whereas the calculated ionic strength of the immersion medium was 0.15 mol/l. Then, the ionic activity product (I_AP_) was calculated for each specific phase and used to calculate the thermodynamic saturation level (S), which is equivalent to the degree of saturation (DS). This parameter represents a direct measure of the thermodynamic driving force for the precipitation of the particular phase. It is calculated as follows:

$$In\frac{I_{Ap}}{K_{sp}}=In\;S$$ (4)

where I_AP_ is the ionic activity product, K_sp_ is the solubility constant at 37°C, and S is the saturation level.

Moreover, a saturation index is defined as follows:

SI= log(S) (5)

## Results

### Material Production and Characterization

Two magnesium based alloys (Mg-2Ag and Mg-10Gd) and the control pure magnesium were used as extruded materials in the study. The chemical composition is presented in Table 1. The results show that levels of Fe and Ni impurities are higher in Mg-2Ag in comparison to the other two materials. Song et al. studied the influence of the impurities on pure magnesium corrosion [38]. According to the tolerance limits which they defined, Fe and Cu were in an acceptable range for our studied materials, whereas the Ni concentration in Mg-2Ag was higher than the defined with 0.0005 wt. % [38].

**Table 1:** The chemical composition of the studied materials.

Alloy	Chemical composition wt. %					
	Ag	Gd	Fe	Cu	Ni	Mg
**Pure Mg**	-	-	0.0042	0.0021	0.0006	Bal.
**Mg-10Gd**	-	10.5	0.0029	0.0048	<0.0036	Bal.
**Mg-2Ag**	2.4	-	0.0048	0.0017	0.0007	Bal.

### Degradation Profile

**Fig 1:**
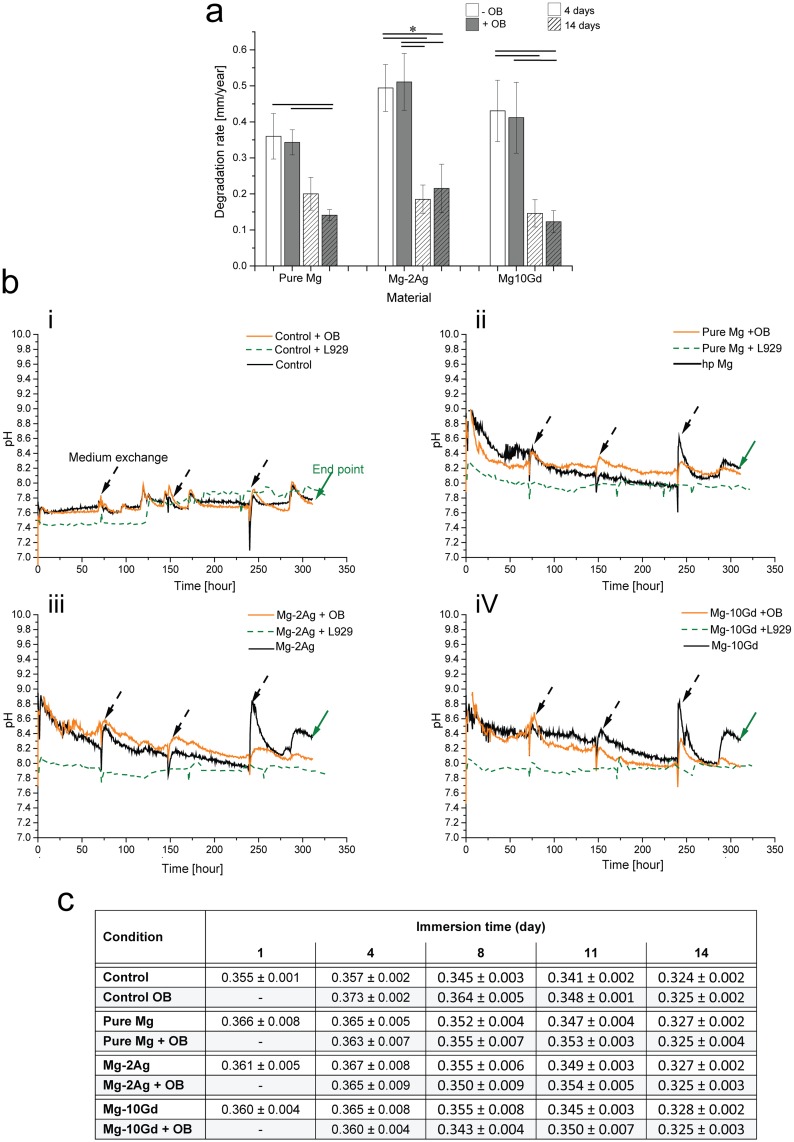
Degradation parameters after 4 and 14 days of immersion with and without cells. a): Mean degradation rate [mm/year] according to the weight loss method for pure Mg, Mg-2Ag and Mg-10Gd discs. Significant differences between the Mg-10Gd and pure Mg were detected using a one way analysis of variance (ANOVA) on ranks with Dunn’s multiple comparison post hoc test at a significance level of p < 0.05. Differences between the conditions of Mg-2Ag were analysed with an ANOVA and the Holm-Sidak post hoc test at a significance level of p < 0.001 (*). b) Online pH measurements during 14 days of immersion. i): controls on tissue culture plastic, ii): pure Mg, iii): Mg-2Ag, and iv): Mg-10Gd. Arrows in the Fig refer to medium exchange and the end point of the immersion test. c): Osmolality changes [Osmole/Kg] during immersion with pure Mg, Mg-2Ag, and Mg-10Gd.

The degradation rates by weight loss after immersion for 4 and 14 days are presented in Fig 1a. The results show the influence of time on the degradation, which is significantly higher for short-term immersion (4 days) than for long-term immersion (14 days) for all materials. The influence of cells was more apparent after 14 days of immersion than after 4 days of immersion. Specifically, a decrease in the degradation rate was observed on the pure Mg (with a reduction factor; 4 d = 95.3%, 14 d = 70.4%) and Mg-10Gd (4 d = 95.6%, 14 d = 84%) compared with the negative control. Conversely, the degradation rate increased when Mg-2Ag was incubated with cells (4 d = 103.4%, 14 d = 116.3%).

During the immersion, pH changes for the different materials were measured online (Fig 1b). Incubation with cells slightly decreased the pH of the controls, which was consistent with the behaviour of L929 cells during the first 125 hours of incubation. The plotted results show that the pH values for all studied materials ranged from 8 to 9. Cells influenced the first 250 hours of immersion depending on the studied alloy. Mg-2Ag with cells showed higher pH values than the negative control (or ‘‘sample no cells” SNC), and this difference was also noted for pure Mg after 72 hours of immersion with cells. However, a contrary effect was observed when Mg-10Gd was incubated with cells. The osmolality (Fig 1c) tended to inversely correlate with the immersion time for all alloys incubated with cells. After 14 days, the osmolality values of experimental samples were in the range of the control value, which indicates a reduction in the dissolving material resulted from the formation of a low-solubility layer on the material surface. Incubation with L929 cells did not significantly change the pH for any of the materials, which can be explained by the natural tendency of these cells to deposit a continuous layer on the material that minimizes the interaction between the material and medium.

### Cell Metabolism with Magnesium-Based Materials

**Fig 2:**
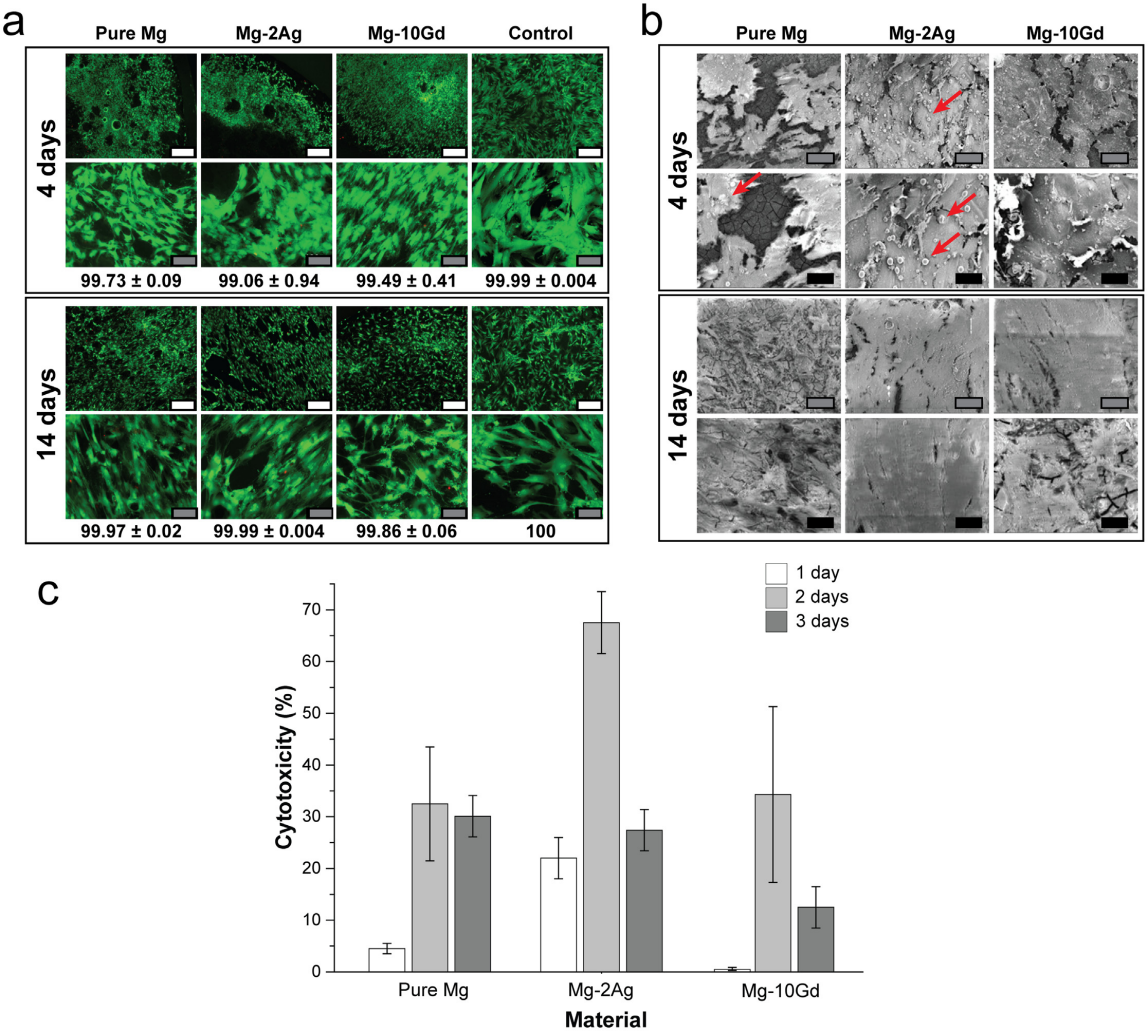
OB cells metabolism in direct contact with pure Mg, Mg-2Ag, and Mg-10Gd. a): Cell viability by Live/Dead staining. Control is cells on tissue culture plates. b): SEM images of cell morphology and coverage. Arrows refer to blebs on the cell surface. c): LDH release from cells induced by the degradation. The measurement was performed after 1, 2, 3, 7, and 14 days of immersion. LDH values at 7 and 14 days (not shown) were approximately 0%. Scale bars in white, grey, and black represent 500, 100, and 50 μm; respectively.

Cells viability was checked by Live/Dead staining (Fig 2a). The results show that the cells were mostly alive. Images obtained after 4 days of immersion at low magnification show a better distribution of the cells on pure Mg and Mg-10Gd than on the Mg-2Ag samples; however, the cells appeared to be more developed and better distributed on all materials after 14 days (Fig 2a). The SEM images (Fig 2b) revealed the cell morphology and adherence on the studied materials. Images of cells obtained after 4 days of incubation on Mg-2Ag revealed cell-surface blebbing. These features were less apparent on pure Mg. In order to check the possible cytotoxic effect induced by the material degradation, Lactate dehydrogenase (LDH) activity in the immersion medium was measured after 1, 2, 3, 7, and 14 days of immersion. At days 7 and 14 the quantified LDH activity was approximately 0%, therefore, only the activities at days 1, 2 and 3 are presented in Fig 2c. After 1 day of immersion, the cytotoxicity of Mg-2Ag was higher than that of the other materials, and this cytotoxicity increased further after 2 days of immersion. This finding indicates the high initial material-medium interaction for this alloy, which induced the found cell damage on SEM images (Fig 2b); however, this influence decreased after 3 days of immersion. On pure Mg, the initial degradation influenced the viability of cells by approximately 5% after one day of immersion; this influence increased to approximately 33% at days 2 and 3 of immersion and decreased thereafter. The influence of Mg-10Gd degradation on cell damage was highest on day 2, with a mean of approximately 34%.

### Mineralization and Cell Distribution

**Fig 3:**
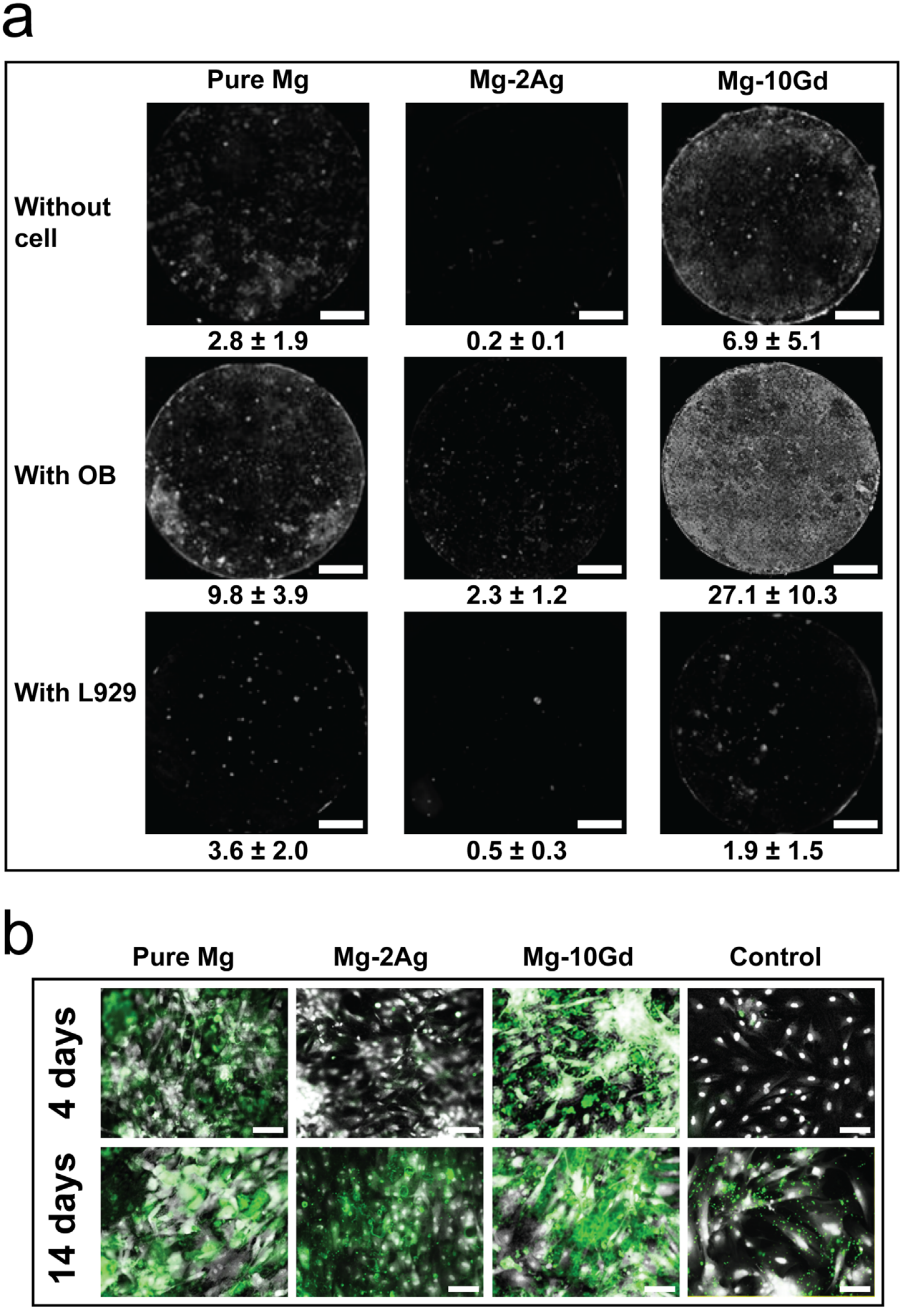
Biominerliazation detection by osteo-Image. a): Fluorescent images of the stained hydroxyapatite (HA) on the different materials in monochromatic mode (i.e., mineralization appears white) after 14 days of immersion; without cells, with primary human osteoblasts, and with L929 mouse fibroblast cell line. The stained area is estimated in % of the shown field of view (values represent the mean of triplicate measurements for each condition). Scale bar represent 2 mm b): Fluorescent images at 20x magnification after 4 and 14 days of immersion. Cells stained with DAPI are shown in white/grey, and the mineralized matrix stained with Osteo-Image is shown in green. Scale bars represent 100 μm.

The influence of cells on altering the chemical composition of the degradation interface by mediating a mineralization process was checked by Osteo-Image, a specific stain for hydroxyapatite (HA). The results obtained for the negative controls (SNC) showed that the medium composition and cell culture conditions synergistically induced the formation of HA, which increased over time (Fig 3a). Specifically, the quantified values were pure Mg = 0.9%, Mg-2Ag = 0.1%, and Mg-10Gd = 0.4% at 4 days and pure Mg = 2.8%, Mg-2Ag = 0.2%, and Mg-10Gd = 6.9% at 14 days. The influence of cells on HA formation is shown in Fig 3a. The amount of HA was highest on Mg-10Gd, followed by pure Mg and lowest on Mg-2Ag. The amount of HA in presence of cells on Mg-10Gd and pure Mg increased by a factor of 4 and approximately 3, respectively. In general, the formation of HA on pure Mg showed a clustered distribution, whereas it was homogeneously distributed over the entire sample surface on Mg-10Gd. The Osteo-Image results of samples with L929 cells were comparable to those of the negative control samples (see Fig 3a).

Fig 3b shows with high magnification multichannel images for DAPI (presented in grey) and Osteo-Image (green). Mineralized matrix and cell distribution was evident on the Mg-10Gd samples after 4 days, and these phenomena were more apparent after 14 days, with overlapping deposits between the cells and HA. However, HA formation induced by cells appeared to be slower on Mg-2Ag, which showed HA staining after 14 days (with stained area of 2.3%) that was comparable to that observed on pure Mg (2.2%) and Mg-10Gd (4%) at 4 days.

### Analysis on the Degradation Interface by SEM/FIB/EDX

**Fig 4:**
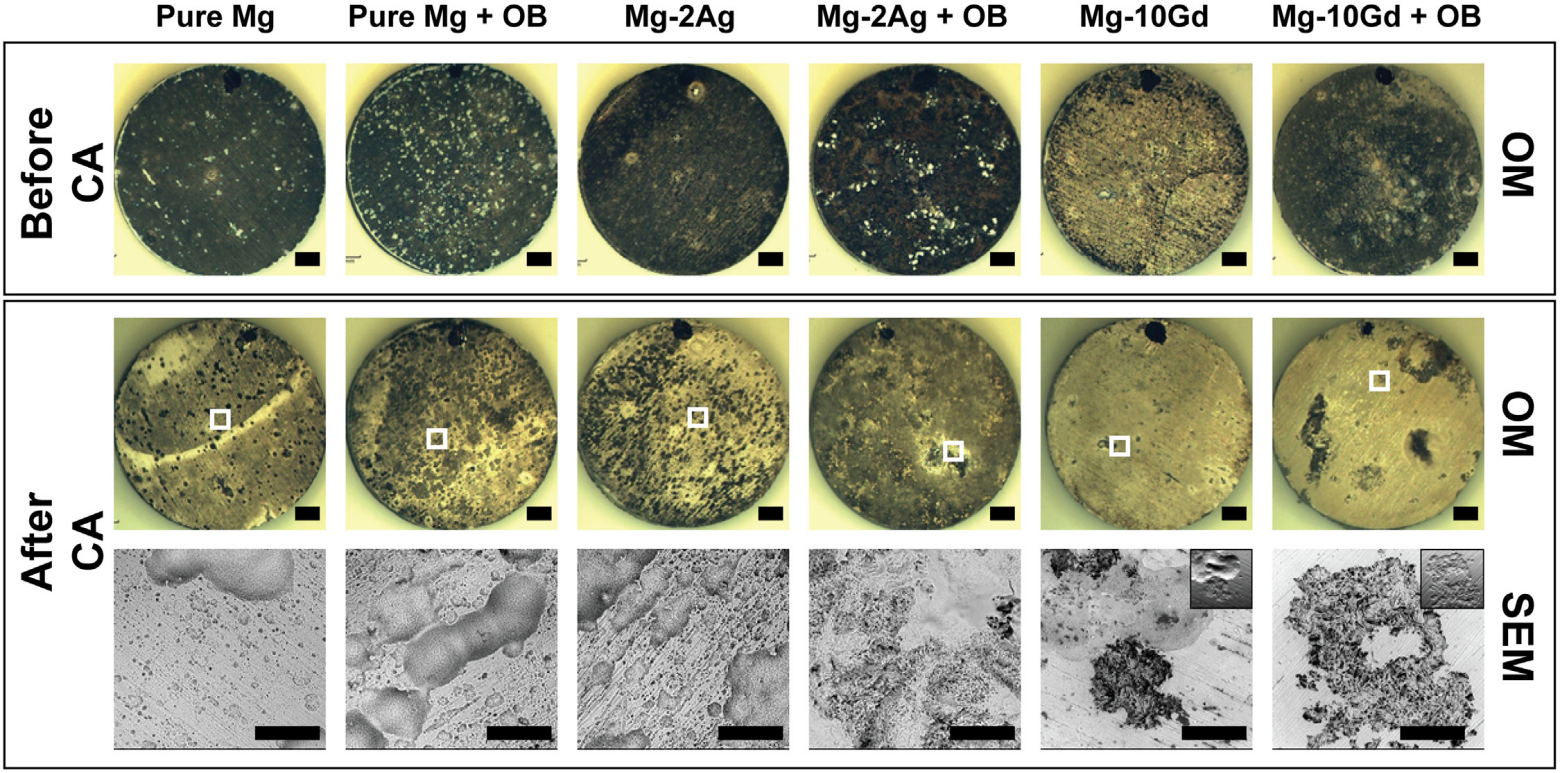
The degradation surface morphology after 14 days of immersion for pure Mg, Mg-2Ag and Mg-10Gd. The figures obtained before and after chromic acid treatment (CA). The black spots on top of each sample are markers to define a coordinated position. Scale bars on these optical microscopic images represent 1 mm. Areas of interests were defined in white frames for further inspection under SEM, where the scale bar on these images represents 200 μm.

The morphology of the degraded material surface before and after chromic acid treatment is shown in Fig 4. The black spots on the surface were identified by SEM to be pits and grooves on the surface due to degradation. The presence of cells on the samples generally increased the formation of these degradation features on the surface (white frames in Fig 4), especially on Mg-2Ag, whose entire surface was covered by these degradation features.

**Fig 5:**
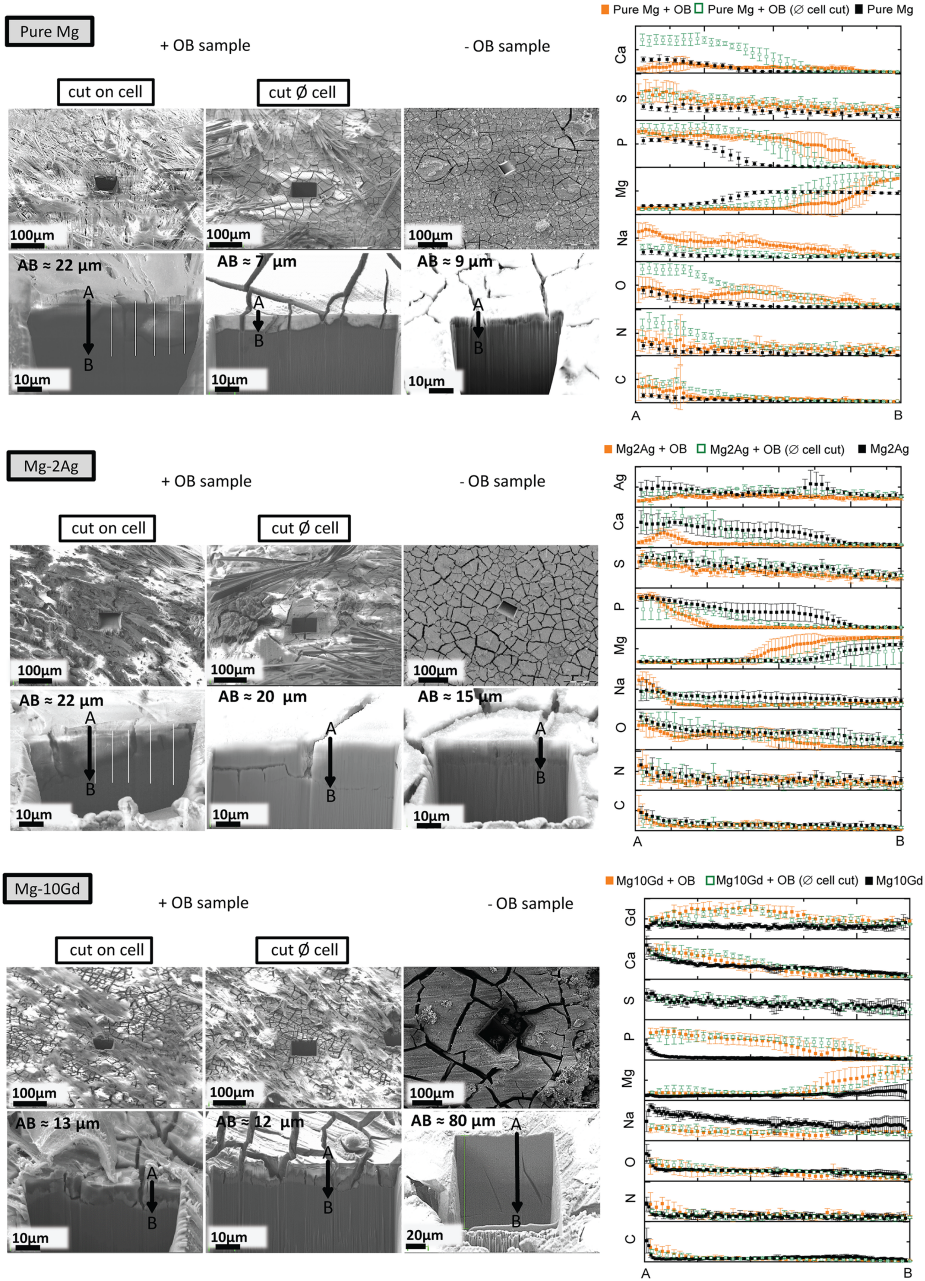
The degradation interface after 14 days of immersion for pure Mg, Mg-2Ag and Mg-10Gd. SEM images of the processed cross sections by FIB of samples without and with OB cells at two positions; on and near the cell. The charts present the EDX line scan measurements on the processed cross sections. |AB| values represent the cross section thickness. EDX line scans start at point (A) and end at point (B).

Fig 5 shows the FIB-processed cross sections of the SNC ‘‘samples no cells” and SC ‘‘samples with cells” at two positions, on and near the cells. SEM images of the cross section were also obtained at high magnification. The degradation layer thickness indicated that the cells significantly influenced the degradation interface of Mg-10Gd: the degradation layer was almost six times thinner than the negative control (SNC) layer, and showed deep cracks with approximately 80 μm in depth. This influence of cells was less pronounced for the other two studied materials. The EDX line measurements of the degradation interface are shown in Fig 5. In this Fig, the results for the different cross sections of the same material are scaled (normalized) to each other and named ‘‘AB” in order to compare the element distribution along the degradation interface in depth for scans performed from the top (point A) to the material bulk (point B). The element quantification of sections directly on cells showed relatively slight increases in S, N, C that coincided with the physical presence of cells. In addition to these elements, pure Mg showed a degradation layer rich in Ca-associated P and O, was observed near cells rather than directly under the cells. This localization can be linked to the degradation morphology of this material in the presence of cells (Fig 4). Specifically, the formation of pits and grooves on the surface can disturb the optimum cell adhesion and result in a non-homogenous influence of cells on mineralization. This Ca-P deposition was also observed on Mg-10Gd in the presence of cells; in this case, the formed degradation layer was rich in Gd (see Fig 5). The EDX scans of Mg-2Ag differed from those of the other two alloys; namely, the levels of Ca and P on the different sections were almost the same, which indicated that cells were minimally active on this alloy, and their role in mineralization was not easily detected with EDX.

## Discussion

### Influence of the Immersion Medium Composition on Ca-PO4 Precipitation

Prior to assessing the influence of osteoblasts cells on degradation by mediating a mineralization process, we found it important to explain the results shown in Fig 3a for the negative samples (samples no cells).

Numerous studies showed that the formation of Ca-PO_4_ is a complex phenomenon that may lead to the formation of different phases, such as dicalcium phosphate (DCP), octacalcium phosphate (OCP), β–tricalcium phosphate (β–TCP), and hydroxyapatite (HA), depending on the experimental conditions. The most important parameters that directly influence precipitation include the following: (1) the temperature, (2) the phosphate and calcium concentrations, (3) the ionic strength, (4) the presence of other interfering ions and macromolecules during the precipitation, and (5) the pH. In our study, the pH changes were measured online throughout the immersion period (Fig 1b). The SNC results demonstrate that the buffering system generally controlled the pH in a range of 8–9. Fernandez et al. studied the solubility isotherms for the Ca(OH)_2_-H_2_PO_4_-H_2_O system at 37°C and found that HA is the most stable phase at approximately pH 8.5, followed by β-TCP [39].

The calculated saturation index indicated that all phases can exist at these specific conditions (Table 2). The most stable phase would be HA, followed by β–TCP, OCP, and DCP. These calculations indicate the precipitation of possible Ca-PO_4_ phases for the medium composition and conditions (i.e., pH and T) used in this study. However, sample degradation will continuously change the surrounding environment, and this change is likely very heterogeneous. For example, the pH may be higher near the material surface, which may affect the favourability of the precipitation of a specific phase over another. Moreover, we did not consider the influence of the dissolved Mg and other alloying elements from the samples, which can change the solution and either increase or reduce the nucleation and growth of different phases. For example, monovalent ions, such as HCO^3-^, Na^+^ and Cl^-^, can reportedly influence the nucleation and growth of precipitated apatite, as indicated in many studies in which amorphous apatite was detected in various biological tissues. This apatite exhibited poor crystallinity and was nonstoichiometric, which was explained by the presence of a significant amount of foreign ions. Some of these ions enter the apatite crystal lattice, whereas others are only adsorbed to the apatite surface [40–43]. Concerning the alloying elements in this study, gadolinium is classified as a Lanthanide element which mentioned to associate with bone [44–47]. This was shown in our results (see Fig 5) where Gd is associated with Ca and P precipitation, whereas silver was dissolved mainly in the media (Figs 3a and 5). One important factor concerning silver is the presence of chloride in the immersion medium (in DMEM 117.5 mM) because these ions proved to increase silver dissolution [48].

**Table 2:** Saturation level index for different Ca-PO_4_ phases according to the immersion medium parameters.

**Solution parameters**			
Ionic strength 0.15 [M]	Temperature 37°C	Ca/PO_4_ 1.97	at pH 8.3
**Saturation level (S)**		**K**_**sp**_ **at 37°C**	**SI = log(S)**
HA: $({\text{Ca}}^{2+}){\;}^{5}\left({\text{OH}}^{-}\right){\left({\text{PO}}_{4}^{-3}\right)}^{3}/{\text{ K}}_{\text{HA}}$		7.36 × 10^−60^	26.03
β–TCP: $({\text{Ca}}^{2+}){\;}^{3}{\left({\text{PO}}_{4}^{-3}\right)}^{2}/{\text{K}}_{\text{TCP}}$		2.83 × 10^−30^	12.36
OCP: $({\text{Ca}}^{2+}){\;}^{4}\left({\text{H}}^{+}\right){\left({\text{PO}}_{4}^{-3}\right)}^{3}/{\text{ K}}_{\text{OCP}}$		1.05 × 10^−47^	8.36
DCP: $({\text{Ca}}^{2+})\;\left({\text{PO}}_{4}^{-3}\right)/{\text{ K}}_{\text{DCP}}$		9.54 × 10^−8^	0.30

### The Interaction: Material Degradation—Cell Metabolism

**Fig 6:**
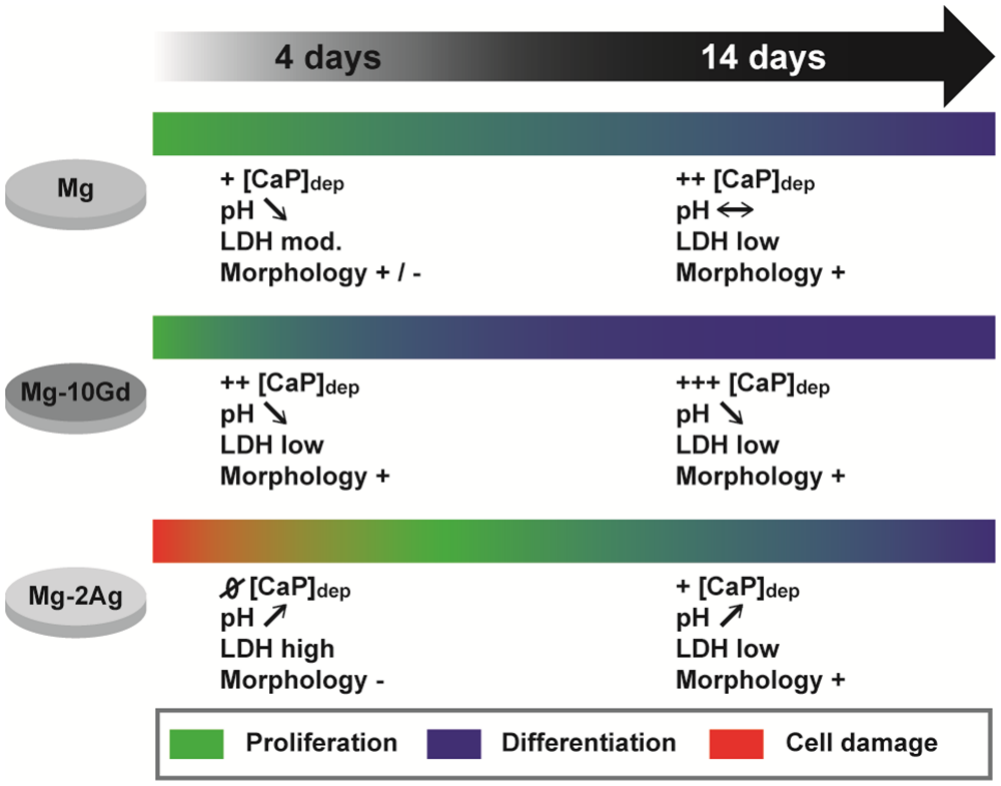
Recapitulation of all the observations on pure Mg, Mg-2Ag, and Mg-10Gd with direct OB contact at 4 and 14 days of immersion. Calcium-phosphate deposition ([CaP]_dep_), pH, LDH activity and cell morphology, and their correlation with cells metabolic activity are presented.

The influence of primary human osteoblasts on the degradation interfaces of Mg-2Ag, Mg-10Gd, and pure Mg was studied. Osteoblasts clearly play a role in bone formation by inducing and regulating the mineralization of the extracellular matrix, whose inorganic component is based on hydroxyapatite [29, 30]. However, the presence of a degradable implant can influence this natural function of osteoblasts. Recent work investigated the influence of pure magnesium degradation on primary human osteoblasts in a direct contact test for four weeks in differentiating medium. Specifically, osteocalcin, osteopontin and bone sialoprotein were up-regulated, which clearly indicates an active mineralization process. When this experiment was carried out with titanium samples, the gene expression levels of these proteins were lower, which revealed that magnesium itself influenced osteoblasts and induced osteoinductive properties [49]. These findings suggest a mechanism by which the cells in our study changed the chemical composition of the degradation interface, although osteogenic differentiation factors were not used in our medium. Alternatively, the metabolic activity of the cells may affect the degradation interface because this activity is combined with the formation and release of lactate into the surrounding environment. This action can negatively influence the degradation of magnesium materials by lowering the pH. Kanan et al. studied the influence of a L929 fibroblast-derived cell line on Mg-Ca alloy. The maximum immersion time was 48 hours. The study showed that pH values were lower when samples were immersed with cells than when samples were immersed without cells. This difference was explained by the influence of lactic acid, which is produced during cell metabolism; consequently, an increase in the degradation rate was observed [50]. L929 cells were included in our study as a control and to assess the specificity of the influence of the materials on the OB metabolic activity and vice-versa. As shown in Fig 1b, the pH values were lower for L929 samples than for OB samples during the first 125 hours, indicating the particularly high initial proliferation rate of L929 cells compared with OB. As a result of this rapid proliferation, more lactate was formed, which decreased the pH. We made the following observations which are recapitulated in Fig 6 for each studied material by closely assessing the material / OB interaction at the degradation interface.

#### Pure-Mg

The LDH activity in the medium was low during the first day of immersion, which suggests that the reactivity between the material and medium was initially low; however, the cytotoxicity subsequently increased on days 2 and 3 and then decreased thereafter, which indicated that the cells tolerated degradation influence. This high initial metabolic activity within the first days of immersion was combined with low pH values for the SC sample compared with the negative control (SNC, see Fig 1b). The pH of the SC sample was maintained at approximately 8.3 thereafter. In addition to their role in mineralization, OB can adhere and extend to form a cell layer that might decrease the interaction between the material and medium and consequently control or even reduce the pH. The SEM images of pure magnesium samples after 14 days of immersion show a non-continuous cell layer, which likely resulted from the mentioned degradation morphology of the magnesium samples. Those findings on the surface morphology corroborated the EDX line measurements of the SC cross sections; specifically, the influence of cells on mineralization was non-homogenous and resulted in a layer richer in Ca and P near the cell than underneath the cells.

#### Mg-2Ag

Fig 1a shows the high initial degradation rate of Mg-2Ag which might resulted from the relatively high concentration of impurities (Table 1). One can assume that this initial high material degradation is combined with Ag^+^ release into the immersion medium. The LDH activity measurements show the influence of this initial reactivity on cytotoxicity, which was high during the first two days of immersion compared with pure Mg and Mg-10Gd; nevertheless, the LDH activity subsequently decreased as immersion continued. The SEM images of Mg-2Ag after 4 days of immersion corroborated the cytotoxicity results showing blebs indicating cell stress or damage (blebbing), which, not surprisingly, lead to less HA deposition, as shown in Fig 3 and the EDX line measurement. It has been previously reported that bleb formation is not only a hallmark of apoptosis or necrosis but also cells injured by physical or chemical stress [51, 52]. These cytotoxic effects seemed to delay OB effect on mineralization, as shown in Fig 3 and the EDX line measurement. Moreover, cells on this alloy negatively impacted the degradation process (Fig 1a), as evidenced by an increase in the pH combined the increase in material degradation. Many studies show that silver nanoparticles are cytotoxic in different *in vitro* and *in vivo* systems [53, 54]. Albers et al. concluded that the cytotoxicity to both primary mouse OB and murine osteoclasts (OC) is primarily mediated by silver ion release. Their study also described the negative effect of Ag^+^, which decreased the differentiation and viability of OB and OC [55]. These findings were consistent with a study by Contreras et al., who used Ag(NH_3_)_2_F salt as a donor for Ag^+^ ions, which was cytotoxic to mouse MC3T3-E1 osteoblasts. Specifically, Ag(NH_3_)_2_F induced cell death after 24 h in 50% of the cells at a concentration of 0.0096 mM, which is almost 5 times lower than the concentration reported by Albers [56]. These differences in the silver concentration tolerated by the cells are attributed to differences in the analytical assays, the used silver salts, and the cell cultures systems. For example, other studies of different human cells showed that silver induced cell cycle progression, DNA damage, and apoptosis. However, at specific low concentrations, the cell proliferation, mitochondrial activity, and cell viability were not affected [57]. This can explain the initial cell damages observed on the Mg-2Ag alloy, which might then followed by cell proliferation rather than differentiation, as evidenced by the mineralization shown in Figs 3 and 5.

#### Mg-10Gd

The potential effects of Gd on bone metabolism are not well understood. Zhang et al. studied the effect of GdCl_3_ on the proliferation, differentiation and calcification of primary mouse osteoblasts *in vitro* [58]. The effect of Gd on bone metabolism was found to be complex, and the concentration and culture time are key factors for assessing the biological role of Gd. Wang et al. studied the influence of Gd complexed with inorganic polyphosphate (polyP), for this concern they compared the influence of polyP.Gd with polyP and GdCl_3_ on hydroxyapatite formation in SaOS-2 cells *in vitro*. Those compounds were found to be non-toxic at concentrations up to 30 μM. At low concentration 5 μM it was found that polyP.Gd and to a smaller extent also polyP or GdCl_3_ caused HA crystal formation arranged in a nest-like shape, which suggested that Gd^3+^ cause an initiation of a differentiation pathway for osteoblasts, with a sequential expression of specific osteoblast markers [59]. Those findings can be correlated to the results in our study where Mg-10Gd degradation was minimally cytotoxic to OB after one day of immersion (Fig 2c). The low pH values after 3 days of immersion compared with the negative control indicated the high metabolic activity of OB (Fig 1b). The results in Fig 3 show an increase in and the homogenous distribution of mineralized matrix precipitation in presence of cells. This observation corresponds with the EDX measurements of the cross sections, i.e., the degradation interface near and underneath the cells exhibits associated Ca, P contents (see Fig 5).

## Conclusion

In summary, the degradation interface with direct cells interaction was successfully analysed by processing micro-sized sections by FIB milling and then subjecting the processed sections of the degradation layer to EDX line scans. Osteoblasts alter the degradation interface actively with their metabolic activity and their innate role in mediating bone formation, and passively by their adhesion and cell layer formation. However, we found that this cells influence was initially influenced by the material degradation rate and morphology. The interface of Mg-10Gd was rich in Ca, P and Gd, which suggests that Gd itself potentially increased HA formation, and contributed to its precipitation. Conversely, Mg-2Ag samples initially exhibited higher cell damage or stress and hence less mineralization on the surface. Pure magnesium revealed moderate cell damage and mineralization compared with the other two studied alloys.
